# Diabetes and tuberculosis syndemic in India: A narrative review of facts, gaps in care and challenges

**DOI:** 10.1111/1753-0407.13427

**Published:** 2023-06-08

**Authors:** Raju Vaishya, Anoop Misra, Abhishek Vaish, Sujeet Kumar Singh

**Affiliations:** ^1^ Department of Orthopaedics and Joint Replacement Surgery Indraprastha Apollo Hospitals New Delhi India; ^2^ Fortis‐C‐DOC Centre of Excellence for Diabetes Metabolic Diseases and Endocrinology New Delhi India; ^3^ National Diabetes, Obesity and Cholesterol Foundation (N‐DOC) New Delhi India; ^4^ Diabetes Foundation (India) (DFI) New Delhi India; ^5^ National Centre for Disease Control (NCDC) Delhi India

**Keywords:** challenges, diabetes, gaps, India, syndemic, tuberculosis

## Abstract

Both diabetes mellitus (DM) and tuberculosis (TB) are prevalent all across in India. TB‐DM comorbidity has emerged as a syndemic and needs more attention in India considering gaps in screening, clinical care, and research. This paper is intended to review published literature on TB and DM in India to understand the burden of the dual epidemic and its trajectory and to obtain perspectives on the gaps, constraints, and challenges in care and treatment of this dual epidemic. A literature search was carried out on PubMed, Scopus, and Google Scholar, using the key words ‘Tuberculosis’ OR ‘TB’ AND ‘Diabetes’ OR ‘Diabetes Mellitus’ AND ‘India’, focusing on the research published between the year 2000 to 2022. The prevalence of DM is high in patients with TB. Quantitative data on the epidemiological situation of TB/DM in India such as incidence, prevalence, mortality, and management are lacking. During the last 2 years convergence of TB‐DM syndemic with the COVID‐19 pandemic has increased cases with uncontrolled DM but also made coordinated control of TB‐DM operationally difficult and of low effectiveness. Research regarding TB‐DM comorbidity is required in the context of epidemiology and management. Detection and bidirectional screening are aggressively warranted. Management of DM in those with TB‐DM comorbidity needs more efforts, including training and supervision of frontline workers.

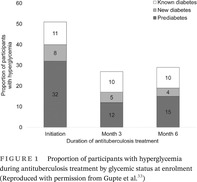

## Graphical abstract

Highlights

- Some data suggest that coexistence of diabetes mellitus and tuberculosis substantially increases the failure of treatment of antituberculosis drugs.
- The COVID‐19 pandemic has severely affected the healthcare delivery of tuberculosis‐diabetes mellitus patients.
- The tuberculosis‐diabetes mellitus syndemic needs a more aggressive focus to have a better outcome.

## 1 INTRODUCTION

Both type 2 diabetes (T2DM) and tuberculosis (TB) are prevalent in India. Prediabetes and T2DM are increasing in urban and rural India at a similar pace^1^ due to rapid changes in lifestyle and physical inactivity. Although T2DM is predominantly urban, it is increasing in semiurban and rural areas as well. Indians develop T2DM at a younger age as compared to whites.^2^ Furthermore, there is an accelerated conversion from prediabetes to DM. The burden of complications, both macro and microvascular, is substantial but varies according to the type of population and regions of India.^3^ During COVID‐19, patients with T2DM presented with higher magnitudes of hyperglycemia, and also new‐onset DM is being seen because of COVID‐19.^4^, ^5^ This has happened due to restriction of activities and poor diets.^6^ The presence of DM leads to a heightened tendency for infection and consequent worsening of hyperglycemia.^5^ It is well known that presence of any infection leads to worsening of hyperglycemia, and this is true for COVID‐19 too. This may be as a result of cytokine storm, stress, or corticosteroid therapy.^7^

India is reported to have the highest burden of TB globally, with 2.4 million cases reported in the National TB Program of India in 2019.^8^ It is possible that some patients with TB were not notified or management was interrupted due to the diversion of the medical workforce to COVID‐19 services.^9^ During the past 2 years, India has been affected simultaneously by COVID‐19, TB, and DM (henceforth termed as TB‐DM; “syndemic”), and although COVID‐19 has potentially waned, TB and DM continue unabated.

This paper is intended to review published literature on TB and DM in India to understand the burden of the dual epidemic and its trajectory and to obtain perspectives on the gaps, constraints, and challenges in care and treatment.

## 2 METHODS

We carried out a literature search on the platforms of PubMed, Scopus, and Google Scholar, using the keywords ‘Tuberculosis’ OR ‘TB’ AND ‘Diabetes’ OR ‘Diabetes Mellitus’ AND ‘India’ from 15 September to 5 October 2022. Since the first case in India was reported from 2002 onwards, all the studies of TB‐DM that have been published in last 2 decades were searched in relation to TB‐DM epidemiology, comorbidity, screening, management, and control programs and the relevant ones were included in this review. We have included 3 systematic reviews, 13 narrative reviews/editorials, 32 original studies, and 16 guidelines/ documents in this review.

## 3 RESULTS AND DISCUSSION

### 3.1 Epidemiology of diabetes and TB globally and in India

#### 3.1.1 Tuberculosis

TB is widely prevalent, present in all age groups, and is present in most countries. In 2019, 2.9 million missing people with TB (“missing millions”) were not reported to national TB programs across the world, due to multiple factors: limited accessibility, underdeveloped health reporting systems, and linkages between public and private sectors as well as lack of human resources. Furthermore, in 2020, there 24% global gap (“missing millions”) between estimated TB incidence and number of newly diagnosed and reported cases of TB, and these numbers are likely to be high in India. With the emphasis on finding the missing cases of TB,^10^, ^11^, ^12^ a number of initiatives have been launched for active case findings in a number of low‐ and middle‐income countries, including India.^10^, ^11^, ^12^ Even with a rapid epidemiological transition in recent years, TB remains one of the top five causes of disability‐adjusted life years as per global burden of diseases data for 2019.^13^, ^14^, ^15^ It is reported to be the 13^th^ leading cause of death and the second leading infectious disease after COVID‐19 to cause mortality in 2020.^10^ The incidence of TB is falling globally, around 2% per year and there has been a cumulative reduction of 11% between 2015 and 2020.

Of note, two thirds of the total cases were present in eight countries, with India at the top of this list (Table 1).^14^ Indeed, the number of incident cases in India is three times more than the next country on the list, China. India is in the middle of an epidemic of TB, with 26% of the total number of global cases, and an estimated incidence of 2.64 million cases in 2019 of which the national program notified 2.4 million.^8^ The newly diagnosed TB infections have risen in India from 1.2 to 2.2 million between 2013 and 2019, with an increase of 74%. The deaths related to TB in India are nearly 0.44 million. Multidrug resistant TB (MDR‐TB) is highly prevalent in India, with one third of global cases.^10^, ^17^, ^18^ Although data notification is built into the national TB control program, more efforts are needed to reproduce the exact number of cases of TB and its incidence, prevalence, and mortality.

**TABLE 1:** Incidence of TB in the top eight countries in 2020.

S. No.	Country	Incidence of tuberculosis	Rate per 100 000 population
1	India	2 590 000	188
2	China	842 000	59
3	Indonesia	824 000	259
4	Philippines	591 000	539
5	Pakistan	573 000	259
6	Nigeria	452 000	219
7	Bangladesh	360 000	218
8	South Africa	328 000	554

*Note*: Adapted from https://tbfacts.org/tb‐statistics/.^16^

#### 3.1.2 Diabetes

In 2021, the largest numbers of adults with diabetes were in China (140.9 million/population), followed by India. However, the countries that have the highest number of people with DM do not necessarily have the highest prevalence.^19^ It is estimated that in 2021, India had 74.2 million diagnosed and undiagnosed adult patients with DM (between 20 and 79 years) and these are likely to rise to 124.9 million in 2045. Alarmingly, there are around 39.4 million (53.1%) undiagnosed cases present in India.^19^

Increasing numbers of patients with DM in India has been evident over the last 2 decades. In a pooled analysis of 1 778 706 adults in India, it was reported that the prevalence of DM has increased in both rural and urban India (from 2.4% and 3.3% in 1972 to 15.0% and 19.0% respectively in 2015–2019). A similar increasing prevalence was also observed for prediabetes. Between 1999 and 2002, the prevalence of prediabetes was 6.0% in rural India, and between 1995 and 2004, its prevalence in urban India was reported to be 11.0%. Between 2005 and 2009, the pooled prevalence of prediabetes in rural and urban India was at 8.0%, and 12.0%, respectively.^19^ Subsequently (2010–2014), the rural prevalence increased to 14.0%, whereas the urban prevalence rose to 17.0%. This prevalence had further risen to 22% in the rural group and 19% in the urban group, between 2015 and 2019.^1^ The high prevalence of DM is also linked to a high burden on micro‐ and macrovascular complications.^20^

### 3.2 TB‐DM comorbidity

There is a bidirectional relationship between DM and infections, and it is true for TB as well.^5^ It is estimated that 15% of adult TB cases are attributable to DM, which is nearly the same for the HIV‐TB association.^13^, ^19^, ^20^ According to a World Health Organization (WHO) report, in 2020, an estimated 370 000 new cases of TB globally were related to DM. In 2019, just over 15% of people with TB were estimated to have diabetes globally, compared with 9.3% among the general adult population (aged 20–79 years). This equates to about 1.5 million people with TB and diabetes who required coordinated care and follow‐up to optimize the management of both conditions.^18^ No authentic data on the overall number of patients with TB‐DM comorbidity are available from India. TB cases in India were attributable to the following risk factors: undernutrition, alcohol use disorders, tobacco smoking, diabetes, and HIV.^21^

Furthermore, the International Diabetes Federation estimated that the number of cases of DM will increase by about 50% globally between 2019 and 2045, with a median increase of 99% (interquartile range [IQR]: 69%–151%) in high TB burden countries.^22^, ^23^

### 3.3 Prediabetes and T2DM among patients with tuberculosis

In a systematic review, Gautam et al^23^ showed that the prevalence of DM in cases with TB was variable in South Asia (Afghanistan, Bangladesh, Bhutan, Maldives, Nepal, India, Pakistan, and Sri Lanka). They reported a pooled prevalence of DM in TB cases of 21%, varying from 11% in Bangladesh to 24% in Sri Lanka (with India at 22%). New‐onset hyperglycemia with TB was evaluated in a meta‐analysis. Eleven studies were included yielding a total of 677 (27.3%). The pooled unresolved (not reverting back to normal glucose regulation, see section 3.5, “Transient Hyperglycemia in Patients with TB”) new cases of hyperglycemia at the end of follow‐up were 50% (95% confidence interval [CI]: 36%–64%) and the total pooled burden of hyperglycemia at 3–6 months of follow‐up was 11% (95% CI: 7%–16%), with both estimates displaying a high heterogeneity.^24^ In a study from Kerala, routine screening of TB cases for DM using glycated hemoglobin (HbA1c) yielded a large number (44%) of DM cases.^25^

There are several factors associated with TB in India. A household community‐based tuberculosis disease survey conducted targeting 69 054 population from 43 villages of five blocks in a district of South India during 2015–2018 revealed that older age, male sex, low body mass index, diabetes, earlier history of TB, and alcohol use were significantly associated with TB.^26^ Mave et al^27^ screened 1793 participants and found 890 cases of microbiologically confirmed TB. The median age of the participants was 32 years; of these 66% were male. The prevalence of pre‐DM and DM was 33% and 18%, respectively. Of the 162 with DM, 59% were known patients and 41% were recently diagnosed. Prevalence of DM among TB cases aged <25 years was 2%, in 25–40 years, 12%, and in patients >40 years, 45%. In this study, each percentage of increase in HbA1c (odds ratio [OR] 1.42, 95% CI 1.01–2.01) was associated with >1 + smear grade or ⩽9 days to TB detection.^28^ There are regional differences in prevalence of DM in TB cases. In a cross‐sectional analysis of the cohort data under Regional Prospective Observational Research for Tuberculosis‐India Consortium, newly diagnosed TB patients (*n* = 1188) were recruited between 2014 and 2018. In this cohort from south India, the prevalence of DM among TB patients was very high, 39% (95% CI: 36.2% to 41.8%).^29^ Interestingly, household contacts (HHCs) of TB patients have a high prevalence of DM. Of 652 adult HHCs, 175 (27%) had prediabetes and 64 (10%) had DM. Forty (64%) HHCs were newly diagnosed with DM and 48 (75%) had poor glycemic control (HbA1c > 7.0%).^26^ This study emphasizes that all HHCs of TB patients should be screened for diabetes.

### 3.4 Tuberculosis in patients with T2DM

The prevalence of TB in patients with DM is variable. In a study in Pune, among 630 adults approached for screening for TB in patients with DM, 70.5% were having poorly controlled DM (HbA1c > 7%). Although 18% of participants reported any TB symptoms, none of these patients were diagnosed with culture‐confirmed TB.^30^ Data were analyzed from the National Family Health Survey (2015–2016), including 107 575 men (aged 15–54) and 677 292 women (aged 15–49).^28^ These authors reported that among those who self‐reported having DM, a total of 866 men and 405 women (per 100 000) also had TB. On the contrary, among those who self‐reported not having DM the figures were 407 men and 241 women (per 100 000). Furthermore, these authors found that adults from poor families, with low body mass index, low levels of literacy, and who were unemployed had a higher risk of TB‐DM comorbidity.^31^ It is not clear if mycobacterium TB mutates to cause more MDR‐TB in patients with diabetes. In a nested cohort (*n* = 1633) studies of TB patients with and without DM in India, the authors found that phylogenetic reconstruction of all 76 paired Mtb strains showed genetically distinct isolates by participant origin, with 84% pairs acquiring at least one single‐nucleotide polymorphism (SNP) at treatment failure or recurrence. The rate of SNP acquisition did not significantly differ by DM status at either treatment failure per 1 person‐year (PY) for TB‐only vs 1.2 per 1 PY for TB‐DM. But there was a significant difference for recurrence per 1 PY for TB‐only vs 1.27 per 1 PY for TB‐DM. The authors concluded that considerable intrahost Mtb mutation rates were present at recurrence among patients with DM in India.^32^

### 3.5 Transient hyperglycemia in patients with TB

**FIGURE 1:**
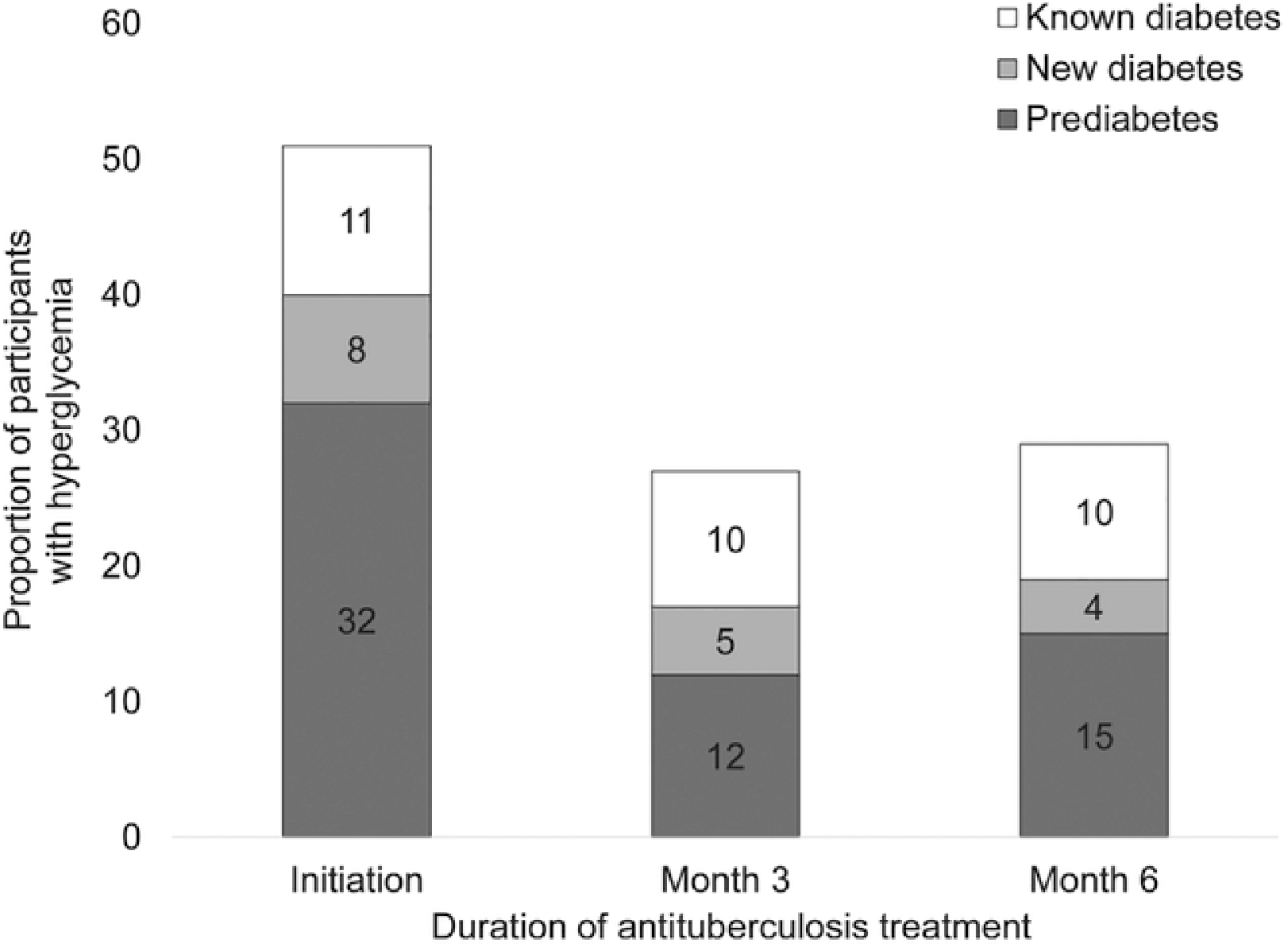
Proportion of participants with hyperglycemia during antituberculosis treatment by glycemic status at enrolment (Reproduced with permission from Gupte et al.^33^)

It is also possible that some patients with TB have transient hyperglycemia that may revert back to normal glucose regulation. Gupte et al^33^ found transient hyperglycemia was common in patients with TB, and HbA1c levels declined significantly during anti‐TB treatment, irrespective of their glycemic status at treatment initiation (Figure 1). These authors suggested that a repeat HbA1c testing should be delayed for at least 3 months from TB treatment initiation to confirm a diagnosis of DM. Second, reversion of HbA1c levels to a nondiabetic range was common in participants with non‐DM. These authors did not provide details of hyperglycemia treatment used by patients recruited in the study. Kornfeld et al^34^ recently reported from south India that the incident TB results in transient glucose elevation but was not conclusively shown to cause chronic dysglycemia. Others have also shown a decreasing trend of hyperglycemia during anti‐TB treatment.^35^

### 3.6 Clinicoradiological presentation of TB in patients with diabetes

Clinicopathological presentation of TB differs in cases with DM vs those without DM (Table 2). Kuruva et al^36^ showed that hemoptysis, weight loss, atypical lung lesions (lower lobe involvement), and cavitations in the lungs were more in those with TB and DM vs TB alone (Table 2). These authors also found lung consolidation to be more common in cases with pulmonary TB associated with DM (68.3%) compared to pulmonary TB without DM (60.3%). Similar findings were observed by Qazi et al.^37^

**TABLE 2:** Differences in clinicopathological presentation and outcomes in patients with tuberculosis with and without diabetes.

Clinicopathological features	Pulmonary tuberculosis with diabetes	Pulmonary tuberculosis without diabetes
Hemoptysis	27%	12.7%
Weight loss	96.8%	84.1%
Lower lung involvement	46%	17.5%
Lung cavitation	42.9%	20.6%
Lung consolidation	68.3	60.3
Sputum conversion (at the end of 2 months)	92.1%	55.6%
Cure rate	61.9%	81%
Treatment failure	14.3%	1.6%

*Note*: Adapted from Kuruva et al.^36^

### 3.7 Treatment outcomes of TB in patients with diabetes

It appears that the overall course of TB‐DM patients is worse than those with TB alone but more data are required. Kuruva et al^36^ showed that sputum conversion and cure rate are lower and treatment failure is higher in TB‐DM patients vs TB alone (Table 2). Mave et al^38^ also showed that early mortality was higher in participants with DM‐TB patients TB alone (10% vs 7%).

In another study 124 patients (cases with diabetes, 68) were recruited. Mortality after therapy was 15% in cases and 7% in controls without diabetes; however, the difference was not statistically significant. Equal proportions in each group (9%) had persistent smear positivity at 2 months.^39^

### 3.8 Pharmacological treatment of TB and DM in patients with TB‐DM comorbidity

Management of DM in patients with TB should be aggressive to prevent failure of anti‐TB treatment. The primary line of treatment in florid TB cases is insulin, but oral antihyperglycemic drugs could be continued with insulin, or even alone if the patient is mildly symptomatic.^40^

The DM affects the effectiveness of anti‐TB drugs through its metabolic effects by impairing the drug metabolism, altering the duodenal efflux pump P‐glycoprotein and pH in the sites of absorption.^41^ Alfrasi et al^42^ showed that patients with TB‐DM had lower concentrations of isoniazid than patients with TB alone, mostly related to higher weight and slower isoniazid absorption. Further, pyrazinamide concentrations were significantly lower in patients with TB‐DM than in those without DM (21.6 vs 32.8 μg/mL), even after adjusting for weight. Finally, these authors also showed that rifampicin concentrations were not affected by DM or HbA1c values, with the *C*_max_ for rifampicin similar in cases with and without DM. Similarly, there were no differences in the rifampicin *C*_max_ by HbA1c category (<5.6%, 5.7%–6.5%, and >6.5%). These authors also stated that suboptimal blood concentrations of these important antitubercular drugs may be an important reason for lower sputum conversion and for treatment failures in patients with TB‐DM vs those with TB without DM. Kumar et al showed a negative correlation between blood glucose and drug concentrations suggesting delayed absorption/faster elimination of isoniazid and pyrazinamide in the presence of elevated glucose.^43^

Usually, physicians resort to insulin if a patient with TB‐DM is suffering from weight loss and fever. Metformin is often stopped. This may not be appropriate given some data from Indian researchers. Mave et al^30^ showed that metformin appeared to mitigate risk of failure of TB treatment and mortality and, therefore, may be important in the management of DM‐associated TB. They have reported that the TB‐DM patients who did not receive metformin in their study had twice the risk of all‐cause mortality, and a six‐time increased risk of death during TB treatment, compared with patients with TB alone. Moreover, metformin reduced the risk of recurrence among patients with TB‐DM.^30^

Further, metformin may have an effect on matrix metalloproteinases (MMPs), which are capable of degrading the pulmonary extracellular matrix. MMP is significantly higher in patients with TB‐DM vs TB. Interestingly, known patients with DM on metformin treatment exhibited significantly lower levels of MMP.^43^, ^44^ Experience with other antihyperglycemic drugs (dipeptidyl peptidase‐ 4 inhibitors, sodium‐glucose cotransporter‐2 inhibitors, and glucagon‐like peptide‐1 receptor analogs) in patients with TB‐DM is limited.

### 3.9 Cost of management of TB‐DM comorbidity

Cost incurred by patients with DM is already substantial.^45^ The research is sparse regarding the cost incurred by the patient if they have DM‐TB. In a study done in the Bhavnagar region of western India, among the 304 patients with TB‐DM comorbidity, 72% were male and the median (IQR) monthly family income was Indian rupees (INR) 9000 (8000–11 000) [~US$ 132 (118–162)]). The median (IQR) total costs due to combined TB‐diabetes were INR 1314 (788–3170 [~US$ 19 (12–47)]), whereas those due to TB were INR 618 (378–1933 [~US$ 9 (6–28)]). The authors concluded that in addition to a marginal increase in the percentage of catastrophic costs, coexistent diabetes nearly doubled the median total costs incurred among patients with TB.^46^

### 3.10 Integrated TB‐DM programs: Gaps and challenges

Although, the Government of India has structured a program for TB control in India, knowledge and practices for diabetes control among primary healthcare workers and physicians may still need to be boosted. The Ministry of Health and Family Welfare in consultation with several strategic experts and stakeholders in TB control has framed a National Strategic Plan for TB 2017–2025.^47^ This comprehensive plan is intended to contribute to the decisions of the policymakers at the national and state levels, development partners, and other stakeholders supporting the Revised National Tuberculosis Control Programme efforts to end TB in India.

Since the WHO initiated an innovative program of “Directly observed treatment, short course” or DOTS in the late 1990s,^46^ there has been a steady increase in the use of this program for TB control services. Since 1995, it is estimated that around 41 million cases have been successfully treated and up to 6 million lives were saved through DOTS. Moreover, 5.8 million TB cases were notified through DOTS programs in 2009.^48^ The WHO and many national TB programs continue to use DOTS as an important strategy for TB delivery for the fear of drug resistance. In 2006 due to several emerging new priority interventions, the WHO then launched the Stop TB Strategy. The new elements of the strategy were represented by targeted measures against MDR‐TB and TB/HIV coinfection, with new attention given to the private sector (up to half of the TB cases are managed in the private sector in the Indian subcontinent), as well as patients and communities. The vision radically evolved in the End TB Strategy launched by WHO in 2014 and consisting of three pillars. The first pillar summarized the core concepts of the previous strategies related to diagnosis and treatment of TB, while introducing for the first time the concept of prevention, mainly based on the identification and treatment of individuals with latent TB infection.^49^

Since 2011, in recognition of the link between TB and diabetes, the WHO has recommended collaborative care for people with TB and DM in the collaborative framework for care and control of these diseases, around the following objectives:

- Establish mechanisms for collaboration;
- Detect and manage TB in patients with diabetes; and
- Detect and manage diabetes in patients with TB.

The key components of the strategy of the Government of India include surveillance of the joint burden of TB and people with DM, and monitoring and evaluation of collaborative TB and DM activities. Routine screening of TB in patients with DM and DM in patients with TB (“bidirectional screening”) provides a unique opportunity for its early diagnosis and better management. This has been outlined in a document from the Government of India.^50^

The following strategies are proposed for collaboration between the National Programme for Prevention & Control of Cancer, Diabetes, Cardiovascular Diseases & Stroke and Revised National Tuberculosis Control Program in India:

- Activities to improve diagnosis and management of diabetes among TB patients:
  - Screening of all registered TB patients for diabetes;
  - Ensuring diabetes management among TB patients.
- Activities to improve diagnosis and management of TB among patients with diabetes
  - Intensified detection of active TB disease among patients with diabetes;
  - Ensuring TB infection control measures in healthcare settings where diabetes is managed; and
  - Ensuring TB treatment and management in diabetes patients.

According to a recent study, gestational diabetes screening should also be prioritized in tuberculosis‐endemic countries, especially in women living with HIV.^51^

Although the TB control part of the program has been in operation for several years and is widely ingrained within health workers, screening and control of diabetes may not be optimal. Screening for TB among DM patients was not implemented, despite documents indicating that it had been. A mixed‐methods study with quantitative (cohort study involving record reviews of patients registered between November 2016 and April 2017) and qualitative (interviews of patients, healthcare providers, and key district‐level staff) components was done in a district of a northern Indian state. Of 562 TB patients, only 137 (24%) were screened for DM. These authors concluded that low patient awareness, poor knowledge of guidelines among healthcare providers, lack of staff, and inadequate training were barriers to screening.^52^ Gurukartick et al^53^ in operational research involving cases with TB‐DM from South India found that the fasting blood glucose was not recorded consistently as per the program guidelines of monitoring at baseline, at the end of intensive, and of continuation phase of TB treatment. These authors also opined that the random blood glucose (RBG) estimation, as done in many cases, is not a reliable indicator of a glycemic control status to analyze the effect on TB treatment outcomes. Second, in the same study, it was noted that a substantial number of patients did not have optimal glycemic control at baseline and during TB treatment but parameters indicating control were not optimal and correct. Specifically, these conclusions are also questionable because it is based on RBG data.

Fazludeen et al^13^ used an outlier case study approach and conducted stakeholder interviews and focus group discussions with relevant program personnel including field staff and program managers of TB and DM control programs as well as officials of partners in Indian states, Kerala (south) and Bihar (north). Several problems were identified (Table 3). Healthcare workers were unable to devote much time to a single program (in this case concerning TB‐DM) because they were part of multiple national programs. In addition, there is a major labor and resource shortage. The disease control programs need to generate more data for better decision‐making and close the skill gaps to improve integrated TB‐DM management.^13^ These authors have suggested decentralization of care and strengthening district‐level coordination (Table 3).

**TABLE 3:** Key suggestions for improvement of TB/DM integrated management.

S. no.	Key message
1	Stronger political commitment to strengthening individual programs
2	Effective integration with general health system through better horizontal‐vertical strategy
3	District level TB/DM program coordination
4	Closing the training and skills gap
5	Resource mobilization
6	Strengthening data systems

*Note*: Adapted from Fazludeen et al., 2022.^13^ Abbreviations: DM, diabetes mellitus; TB, tuberculosis.

But carefully programmed integrated management of TB‐DM is achievable by non‐physician health workers, even in the low resource setting of rural India. In a pilot study (a randomized controlled trial design with mixed‐methods evaluation), conducted in the Guntur district of Andhra Pradesh (South India), 120 newly diagnosed patients with TB and screened for DM were evaluated. Nonphysician health workers were trained to encourage adherence to treatment and monitor treatment response including blood glucose levels and provide lifestyle advice and were considered as intervention arm. Although blood glucose levels in both arms of trial were similar, awareness about DM and TB comorbidity and cardiovascular risk increased in the intervention arm of the study.^54^ Overall, this intevention strategy was well accepted by the patients and healthcare providers. These authors concluded that with appropriate training, availability of infrastructure, and planned intervention implementation, it is feasible to co‐manage TB‐DM within the existing primary healthcare system in India.^54^

Overall, the findings show that although WHO first recommended collaborative activities to address TB and DM in 2011, implementation in India is variable and inadequate in many states and regions. It has been strengthened after a National Framework for Joint TB‐Diabetes Collaborative Activities was laid down. This detailed document lists methods of screening, recording, and reporting of TB‐DM comorbidity at all levels of medical care. In addition, method of blood glucose testing and forms for proper reporting of diabetes in TB centers and TB in diabetes and general medical facilities have been provided. Administrative set‐up and guidance for education and training have been clearly laid down.^55^ However, ground‐level research, studies involving large samples, and prospective cohort data are needed. There are some data available regarding bidirectional screening^13^ and management^56^ but more studies are required. Monitoring and evaluation of the joint response will be critical for driving the scale‐up and for assessing the impact of TB and diabetes collaborative activities. The challenges lie in strengthening the individual programs and envisioning and implementing a horizontal‐vertical integration. The wide network of public health facilities in India offers clear opportunities to improve service delivery.

### 3.11 Impact of COVID‐19 pandemic on DM and TB

During the COVID‐19 pandemic, several studies indicated that patients with DM had increased severity of COVID‐19 and also post‐COVID‐19 syndrome.^56^ It is perhaps due to the effect of DM on the viral entry into the cell and inflammatory response to the infection or the effect of corticosteroid therapy.^13^ Patients with known DM had uncontrolled glycemia, and in addition, new‐onset DM were also seen.^4^, ^57^ Such patients with new‐onset DM add to the existing large numbers of DM patients in India. Further, uncontrolled glycemia would increase the tendency for contracting TB.

It is reported that the history of TB (active or latent) increases the risk and susceptibility for SARS‐CoV‐2 infection. Furthermore, it causes more severe clinical manifestations of the disease and its progression and is associated with poor outcomes. Specifically, TB increases 2.1‐fold risk of severe COVID‐19 disease.^58^ Because diabetes also increases risk of morbidity and mortality in patients with COVID‐19,^59^, ^60^ it is reasonable to suggest a bidirectional TB‐COVID‐19 screening, in addition to screening for diabetes, should be done, that is, COVID‐19 screening (depending on clinical symptoms and signs) for TB cases at diagnosis and TB screening for all COVID‐19 cases.

In addition, COVID‐19 pandemic had a significant impact on the healthcare delivery for various aspects of TB such as prevention, surveillance, and treatment programs.^61^ The following are summary points regarding the COVID‐19 pandemic and TB‐DM syndemic in India:

1. Uncontrolled DM is more common during the COVID‐19 pandemic than before the pandemic^4^
2. Addition of patients with new‐onset diabetes due to COVID‐19 or its therapy (corticosteroids)^57^
3. Suboptimal treatment of DM due to lockdown and decreased physician contact
4. Interruption of national TB program
5. Suboptimal treatment of TB^62^, ^63^

## 4 CONCLUSION

Prevalence of DM and TB is substantial in India. Some studies show that uncontrolled DM increases the risk of treatment failure with anti‐TB treatment. The convergence of the TB‐DM syndemic with the COVID‐19 pandemic has increased cases with uncontrolled diabetes and also made coordinated control of TB‐DM operationally challenging. The TB‐DM syndemic needs a more aggressive focus to have a better outcome of TB treatment. National programs should include bidirectional screening and aggressive control of glycemia in TB‐DM patients.

## FUNDING INFORMATION

No funding is procured for this research from any agency.

## CONFLICT OF INTEREST STATEMENT

None.
